# Construction of a Human Cell Landscape of COVID-19 Infection at Single-cell Level

**DOI:** 10.14336/AD.2021.0301

**Published:** 2021-06-01

**Authors:** Jian He, Yingxin Lin, Mei Meng, Jingquan Li, Jean YH. Yang, Hui Wang

**Affiliations:** ^1^State Key Laboratory of Oncogenes and Related Genes, Center for Single-Cell Omics, School of Public Health, Shanghai Jiao Tong University School of Medicine, Shanghai 200025, China.; ^2^School of Mathematics and Statistics, Charles Pekins Center, The University of Sydney, Sydney, Australia.

**Keywords:** COVID-19, SARS-CoV-2, Single-cell RNA-Seq, ACE2, TMPRSS2

## Abstract

COVID-19 is now causing a global pandemic, there is a demand to explain the different clinical patterns between children and adults. To clarify the organs/cell types vulnerable to COVID-19 infection and the potential age-depended expression patterns of five factors (*ACE2*, *TMPRSS2*, *MTHFD1*, *CTSL*, *CTSB*) associated with clinical symptoms. In this study, we analyzed expression levels of five COVID-19 host dependency factors in multiple adult and fetal human organs. The results allowed us to grade organs at risk and also pointed towards the target cell types in each organ mentioned above. Based on these results we constructed an organ- and cell type-specific vulnerability map of the expression levels of the five COVID-19 factors in the human body, providing insight into the mechanisms behind the symptoms, including the non-respiratory symptoms of COVID-19 infection and injury. Also, the different expression patterns of the COVID-19 factors well demonstrate an explanation that the different clinical patterns between adult and children/infants.

COVID-19, which emerged in late 2019, is now causing a global pandemic [1, 2]. SARS-CoV-2 has been demonstrated as the virous mediating the COVID-19 infection [1-3]. It has been reported that SARS-CoV-2 invades human cells via host dependency factors, including *ACE2*, *TMPRSS2*, *CTSL* and *CTSB*, and these factors are all SARS-CoV-2 receptors [4-8]. In addition to these SARS-CoV-2 receptors, more recently, *MTHFD1*, has been shown to be critical for viral replication [9]. While the lungs are the primary target, the virus can subsequently invade and damage organs, including the heart and blood vessels, kidneys, gut, and brain [3-7], but the cell types vulnerable to COVID-19 infection still unclear. Also, there is a demand to explain the different clinical patterns between children/infants and adult.

To clarify the organs/cell types vulnerable to COVID-19 infection and the potential age-depended expression patterns of five factors associated with clinical symptoms, in this study, we used published single-cell RNA-seq data to analyze expression levels of five COVID-19 host dependency factors in different cell types of multiple human organs both in adults and fetus, including intestine, kidney, lung, liver and heart [10, 11].

Firstly, the bulk RNA-seq analysis results of all organs indicated that *TMPRSS2 and ACE2* are highly expressed in prostate and testis related tissue/cells respectively (Supplementary Fig. 1A, B). *ACE2* also highly expressed in small intestine, trigeminal ganglion, and skeletal muscle. *CTSL* and *CTSB* both highly expressed in placenta, uterus corpus, fetal lung, thyroid and CD71^+^ early erythroid, while *MTHFD1* is highly expressed in thymus, liver of organs and some types of cells (Supplementary Fig. 1D, E). Furthermore, we explored scRNA-seq data from different parts of the adult human digestive system (Supplementary Fig. 2A-H). We found an interesting phenomenon that paneth cells rather than enterocyte, took the largest proportion in adult ascending colon, duodenum, epityphlon and transverse-colon. Enterocyte only dominates in jejunum, rectum and sigmoid-colon parts in adult digestive system (Supplementary Fig. 3A; Supplementary Fig. 4A; Supplementary Fig. 5A). *TMPRSS2* positively expressed in majority cell types in adult intestine except in myeloid and neuron cells. *ACE2* is also expressed in many cell types in adult intestine. *MTHFD1* is expressed with a broader distribution except for conventional DCs, enteric glial cells, myeloid cells. Unlike *CTSB* is expressed in all cell types and highly expressed in immune cells (DCs, macrophage and neuron cells) in adult intestine, *CTSL* did not show any expression in CD8^+^ T cells (Supplementary Fig. 3A; Supplementary Fig. 5A). The expression level of *TMPRSS2* enrichment in inflamed epithelial cells and goblet cells. The *ACE2* expression is enriched in paneth cell and enterocyte, which are the top two proportion cell types in intestine system. The expression level of *MTHFD1* enrichment in proliferating B cell, neuro and enterocyte progenitor. Both the expression level of *CTSL and CTSB* is enriched in stromal cells, neuron, myeloid cells, macrophage, and *CTSB* is also enriched in DCs and conventional DCs (Supplementary Fig. 3A). Interestingly, most of these factors were co-expressed in enterocyte and goblet cells which implying enterocytes and goblet cells can be considered as the target of COVID-19 infection make the intestine as the high-risk organ (Supplementary Fig. 3A, B; Supplementary Fig. 6A, B). In fetal intestine (Supplementary Fig. 2 I), a highly expressed *ACE2* level was detected in enterocyte-APOA4 high, a subtype of enterocytes, in adult duodenum and jejunum respectively. Firstly, enterocyte progenitor cells and lymphatic endothelial cells, two new cell types were detected in the fetal intestine donor (Supplementary Fig. 2I; Supplementary Fig. 5A, B; Supplementary Table 2), and goblet cells, enterocytes, proliferating cells demonstrated higher expression level of *ACE2* in the fetal donors the in adult donors (Supplementary Fig. 3A, B; Supplementary Fig. 5A, B). This made fetal intestine also a high-risk organ as the adult intestine, even a higher risk grade, but via different infection targets, rather than paneth cell and enterocyte (in adult). Secondly, we also found that these factors were enriched in antibody-related cells, such as B cells and antigen-presenting cells, which may be implying fetal demonstrate a different immune response to the infection (Supplementary Fig. 3A, B; Supplementary Fig. 6B).

We also analyzed the scRNA-seq data from the kidney of urinary system from three adult and four fetal donors. *TMPRSS2* expression enrichment in intercalated cells was detected both in adult and fetal donors, while IC-tran-PC cells only with high enrichment of *TMPRSS2* in adult donors (Supplementary Fig. 2J, K; Supplementary Fig. 3C, D, Supplementary Fig. 4B, G; Supplementary Fig. 6C, D). The expression *ACE2, MTHFD1, CTSL and CTSB* are all enriched in proximal tubule cells. Both *CTSL* and *CTSB* are highly enriched in myeloid cells, macrophage, and *CTSB* also enriched in epithelial cells, DCs, conventional DCs and B cells. The co-expression analysis illustrated that proximal tubule cells and intercalated cells might be with higher co-expression of the combination of the factors. Moreover, proximal tubule cells and intercalated cells are the main part of the cell population (Supplementary Fig. 4; Supplementary Fig. 6). Therefore, the proximal tubule cells and intercalated cells could be considered as targets which makes adult kidney at high risk (Supplementary Fig. 5C; Supplementary Fig. 6C, D). Compare with adult kidney, besides enriched in intercalated cells and distal tubule progenitor cells, *TMPRSS2* also enriched in ureteric epithelial cells (Supplementary Fig. 5C) in fetal kidney (Supplementary Fig. 4G; Supplementary Fig. 5D; Supplementary Fig. 6D). Also, higher *ACE2* expression was found in proximal tubule progenitor cells, while *TMPRSS2* and *MTHFD1* only positive expressed in other cell types. This is a different receptor and cell types of expression pattern in fetal kidney (Supplementary Fig. 5D, Supplementary Fig. 6D). Finally, B cells highly expressed *CTSB* in adult kidney while there was no B cell was detected in the fetal kidney (Supplementary Fig. 4 B, G; Supplementary Fig. 5D; Supplementary Fig. 6D). The co-expression analysis showed proximal tubule cells are the target of the virus attraction both in the adult and fetal kidney (Supplementary Fig. 5D; Supplementary Fig. 6D).

*ACE2* expression was generally low in all three adult and two fetal lung donors (Supplementary Fig. 2L-M; Supplementary Fig. 5E, F). Very few ACE2 positive cells were detected neither in adult nor in fetal lung tissue. Moreover, the expression of ACE2 level in AT1 cells, which has been described as the “putative mechanism” of the lung infection, is quite low in adult donors, and we neither found any AT1 or AT2 cells in fetal donors (Supplementary Fig. 4C, H; Supplementary Table 2). In contrast, high *TMPRSS2* expression was detected in AT1, AT2 and other cell types in adult lung (Supplementary Table 2), while the *TMPRSS2* expression could not be detected in most cell types in fetal lung (Supplementary Fig. 5E, F). Positive *TMPRSS2* expression was found in all cell types except in megakaryocyte and myeloid cells, and positive *MTHFD1* expression was found in all cell types except bronchial chondrocyte, ciliated cells, and stromal cells in adult lung. *CTSL* and *CTSB* are both expressed in all cell types in adult lung (Supplementary Fig. 3E; Supplementary Fig. 5E). There was no cell co-expressed *ACE2* or any other factors, while distal progenitor cells co-expressed *TMPRSS2* and other factors, such as *MTHED1*, *CTSL* and *CTSB*. Also, the co-expression of *MTHFD1-CTSL* and *MTHFD1-CTSB* was detected in many cell types in the adult lung (Supplementary Fig. 6). All these indicate that *ACE2* may not be the key factor (or the only factor) of the COVID-19 infection, the high expression of other factors (even some other cofactors), such as *TMPRSS2 and MTHFD1* could be considered as the target of COVID-19 infection make the adult lung as the high-risk organ. Also, positive *MTHFD1* expression was detected in various cell types both in adult and fetal lung tissue (Supplementary Fig. 3E, F). Compare with the fetal kidney, the universal expression of *TMPRSS2* and high expression/co-expression level of *CTSL* and *CTSB* in adult lung make the adult lung as the high-risk organ than the fetal. Secondly, the large proportion of lung mesenchyme cells (cardiopulmonary progenitor cells) in adult with positive expression of the factors make adult more vulnerable to virus infection than fetal or infants (Supplementary Fig. 3E, F; Supplementary Fig. 4C, H). Lastly, the factors were co-expressed in the macrophage and megakaryocyte/ erythroid progenitor cell, which implies that immune cells also could be the targets of the virus infection or involved in the cytokine storm leading to the so-called macrophage activation syndrome (MAS) (Supplementary Fig. 5E, F; Supplementary Fig. 6E, F).

**Figure 1.**
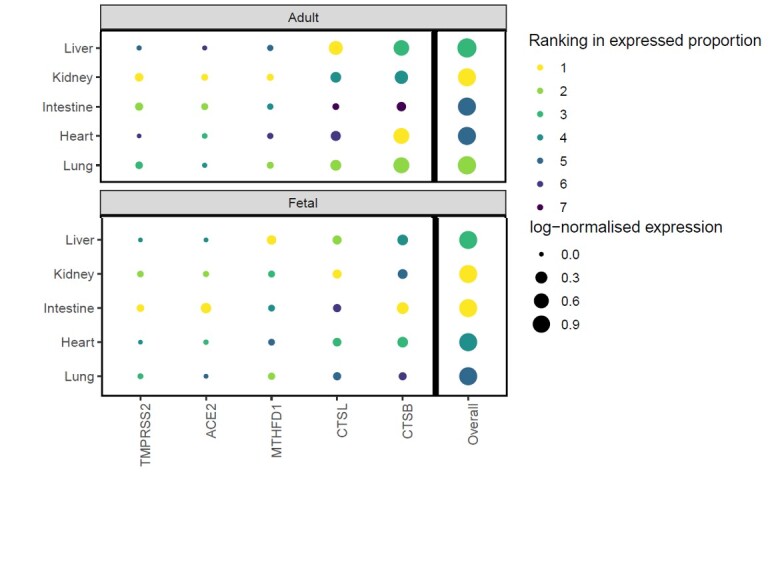
Grade adult and fetal organs at risk. Ranking is the based on the expressed proportion of each gene in each organ. Rank 1 indicates it has highest expressed proportion, that is the highest risk. Overall ranking is the average of the individual ranking.

*MTHFD1* expressed in hepatocytes in three adult liver donors and positive expressed in many immune cell types including neutrophil, effector T cell, monocyte, T cell and so on (Supplementary Fig. 2N, O; Supplementary Fig 3D, I; Supplementary Fig 4D, I; Supplementary Fig. 5M, N), whereas besides in epithelial cells, neither *ACE2* nor *TMPRSS2* was expressed in adult and fetal liver, which implying that liver as a high-risk organ due to the highly expressed *MTHFD1* rather than *ACE2* and *TMPRSS2*. Same as intestine, kidney and liver, the expression of *CTSL* and *CTSB* with a broader distribution in all cell types in liver (Supplementary Fig. 5G, H). The co-express analysis demonstrated that sinusoidal endothelial cells and epithelial cells are the targets leading to adult liver vulnerable to COVID-19 infection (Supplementary Fig. 3G, H; Supplementary Fig. 6G, H). Liver sinusoidal endothelial cells (LSECs) are highly specialized endothelial cells representing the interface between blood cells on the one side and hepatocytes and hepatic stellate cells on the other side. LSECs represent a permeable barrier. This would be a well explanation for the liver failure in adult COVID-19 patients rather than in children/infant patients. Finally, we found that most factors enrichment in endothelial cells and epithelial cells (specific in adult with large proportion) rather than in hepatocyte in adult, which may be indicating that endothelial cells and epithelial cells may be the reason that adult are more vulnerable to COVID-19 infection than infants (Supplementary Fig. 3D, I; Supplementary Fig. 5G, H; Supplementary Fig. 6G, H).

None of the cells expressed *TMPRSS2*, and only a few types of cells, such as fibroblast, cardiomyocyte, neutrophil with the positive expression of *ACE2* in two adult and two fetal heart donors (Supplementary Fig. 2P, Q). Besides immune cells, *CTSL* and *CTSB* also both enriched in ventricle cardiomyocytes, vascular endothelial cells, cardiomyocyte and apoptotic cells (Supplementary Fig. 3I, J). We found that most factors enrichment in ventricle cardiomyocyte (specific in fetal with the largest proportion) and in proliferating cells in fetus, which may be indicating that children/infants could also be victim to COVID-19. And fibroblast is enriched most factors in adult heart, so fibroblast might be the target of the virus leading to adult heart vulnerable to COVID-19 infection (Supplementary Fig. 6I, J).

In summary, the results above allowed us to point the target cell types in each organ, also graded organs at risk (Fig. 1). Overall ranking is the average of the individual ranking. From the overall ranking, we found that the liver and kidney of adult and fetal share same risk value, and kidney is the highest risk to the COVID-19 infection (liver rank value 3 and kidney rank value 1, rank 1 is the highest risk value and rank 7 is the lowest value). Fetal intestine and heart are more likely infected by COVID-19 than that of adult, and fetal intestine like fetal kidney, both of them are top risk to be infected. Adult lung is more vulnerable to infection due to the much higher expression of *CTSL* and *CTSB* and slightly higher expression of *ACE2*. Also, the different expression patterns of the COVID-19 factors (*TMPRSS2*, *ACE2*, *MTHFD1*, *CTSL* and *CTSB*) well demonstrates an explanation that the different clinical patterns between adult and children/infants.

The results allowed us to grade organs at risk and pointed towards the target cell types in each organ mentioned above. A comparison of fetal and adult organs and cell types suggest that low *ACE2* and *TMPRSS2* expression level in various subpopulation of enterocyte cells in fetal intestine, lower *ACE2* expression in various subpopulations of intercalated cells in fetal kidney and lacking *ACE2* expression in most subtypes cells in fetal lung could be major factors determining the well-documented reduced risk of infants.

Based on these results we constructed an organ- and cell type-specific vulnerability map of the expression levels of the five COVID-19 factors (*TMPRSS2*, *ACE2*, *MTHFD1*, *CTSL* and *CTSB*) in the human body, providing insight into the mechanisms hidden behind the symptoms, including the non-respiratory symptoms of COVID-19 infection and injury. Also, the different expression patterns of the COVID-19 factors well demonstrate an explanation that the different clinical patterns between adult and children/infants.

In summary, our study provides an overview of COVID-19 infection-related human vulnerable organs based on single-cell analysis. We first time managed to elucidate vulnerable organs and stratify organs of fetal and adult into high and low risk according to the expression level of COVID-19 receptors, including *ACE2*, *TMPRSS2* and the key enzyme for viral replication, *MTHFD1* in certain cell types. Also, we study the different receptors expression level between adult and fetal. This finding may explain why adults are more likely to suffer COVID-19 infection and the non-respiratory symptoms observed in COVID-19 pneumonia patients, such as diarrhea and multiple organ failure.

## Supplementary Materials

The Supplemenantry data can be found online at: www.aginganddisease.org/EN/10.14336/AD.2021.0301.


