# Supplementary material for: Construction of a Human Cell Landscape of COVID-19 Infection at Single-cell Level
Source: Aging Dis. 2021 Jun 1;12(3):705–9. doi: 10.14336/AD.2021.0301 (PMC8139199; doi:10.14336/AD.2021.0301)
Supplement: Supplementary file 1 [file AD-12-3-705-s.pdf]

SUPPLEMENTARY DATA

Construction of a Human Cell Landscape of COVID-19 Infection at Single-cell Level

Jian He[1#], Yingxin Lin[2#], Mei Meng[1], Jingquan Li, Jean YH. Yang[2], Hui Wang[1*]

© 2021. He J et al. Published online at http://www.aginganddisease.org/EN/10.14336/AD.2021.0301

SUPPLEMENTARY DATA

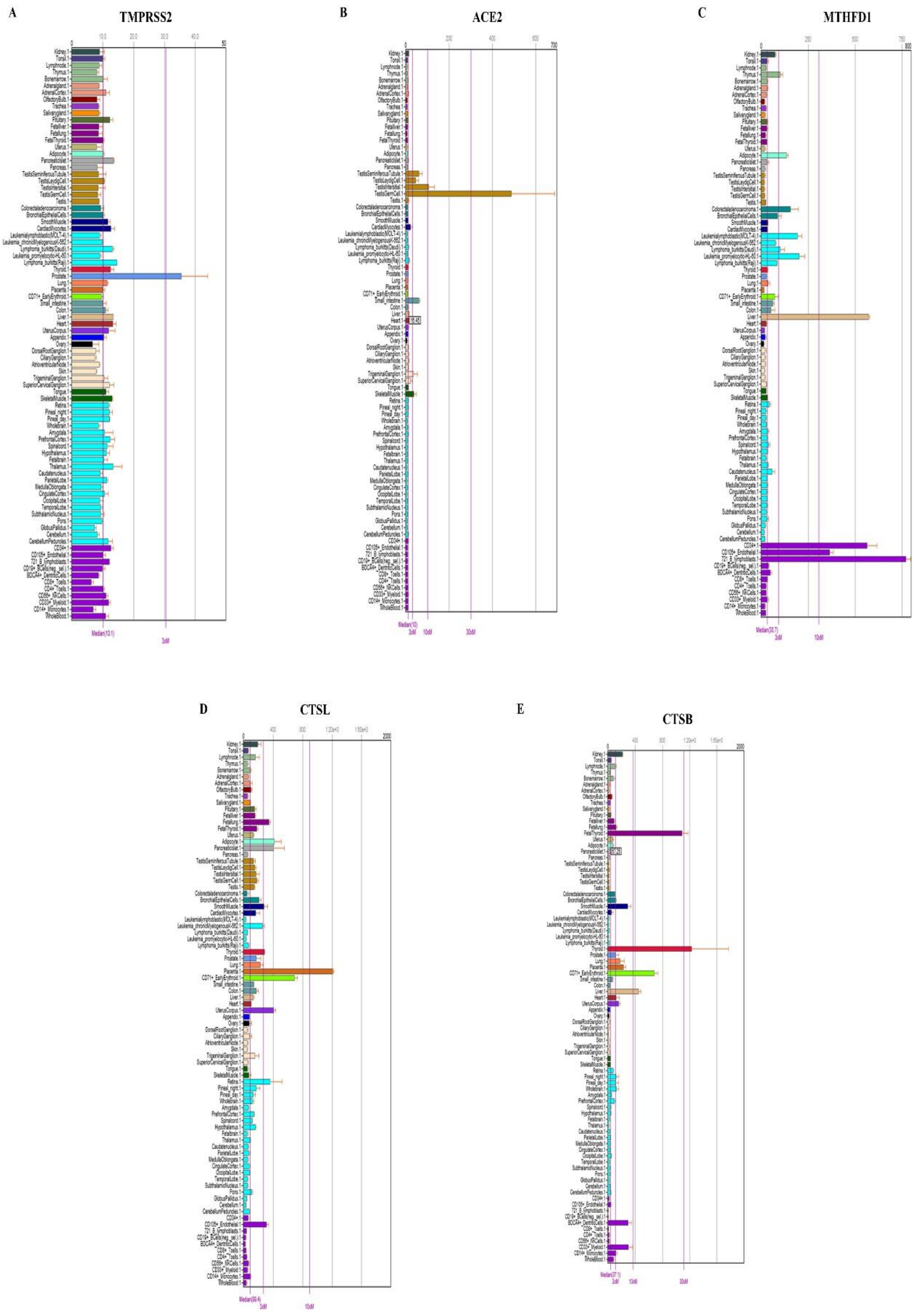

Supplementary Figure 1. Bulk data analysis showed prostate and testis have high *TMPRSS2* (A) and *ACE2* (B) expression level respectively, and liver exhibit high *MTHFD1* (C) expression. Placenta cells and thyroid demonstrated high expression level of *CTSL* **(D)** and *CTSB* **(E)** respectively.

© 2021. He J et al. Published online at http://www.aginganddisease.org/EN/10.14336/AD.2021.0301

SUPPLEMENTARY DATA

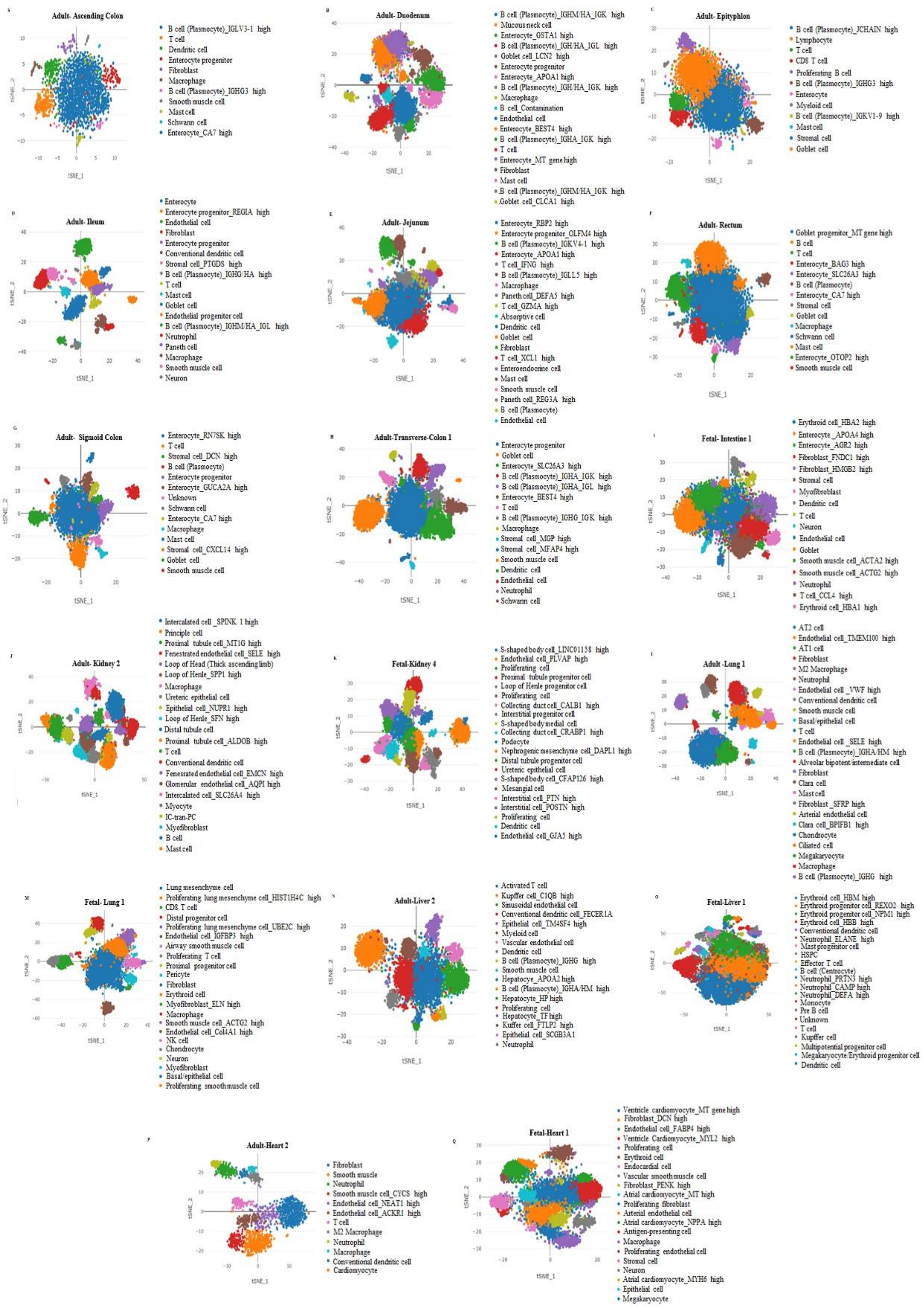

Supplementary Figure 2. ScRNA-seq data analysis showed the cells in different organs were categorized into subclusters. (A) Ascending colon, **(B)** Duodenum, **(C)** Epityphlon, **(D)** Ileum, **(E)** Jejunum, **(F)** Rectum, **(G)** Sigmoid colon, **(H)** Transverse-colon, **(I)** Representative for four fetal intestine donors, (J) Representative for three adult kidney donors, **(K)** Representative for four fetal kidney donors, **(L)** Representative for three adult lung donors, **(M)** Representative for two fetal lung donors, **(N)** Representative for three adult liver donors. **(O)** Representative for two fetal liver donors, **(P)** Representative for two adult heart donors, **(Q)** Representative for two fetal heart donors.

© 2021. He J et al. Published online at http://www.aginganddisease.org/EN/10.14336/AD.2021.0301

SUPPLEMENTARY DATA

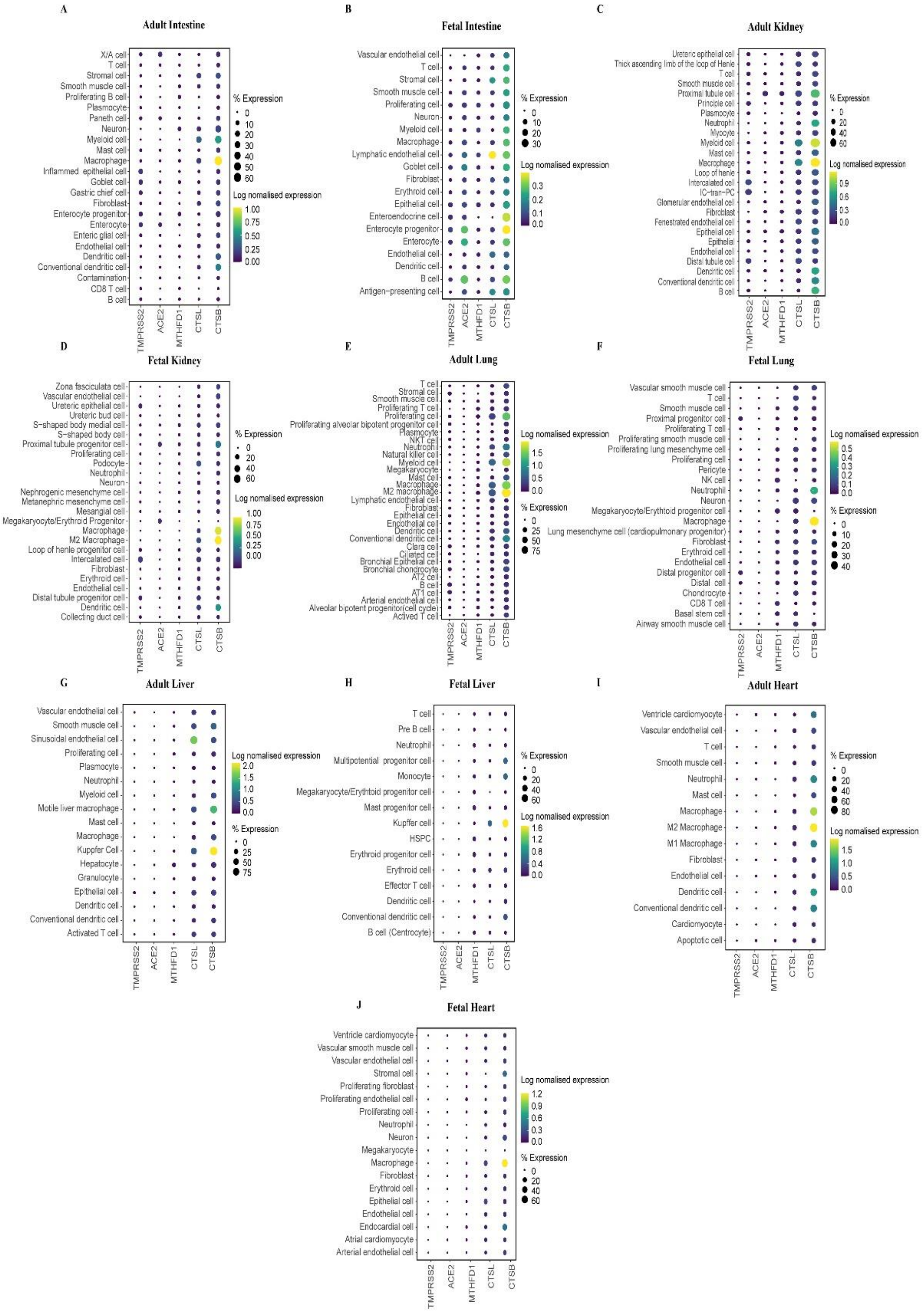

Supplementary Figure 3. ScRNA-seq data analysis showed the cells in different organs of the adult and fetal intestine donors and the grade adult and fetal organs at risk. Enrichment of the five factors in adult (**A**) and fetal (**B**) intestine. Enrichment of the five factors in adult (**C**) and fetal (**D**) kidney. Enrichment of the five factors in adult (**E**) and fetal (**F**) lung. Enrichment of the five factors in adult (**G**) and fetal (**H**) liver. Enrichment of the five factors in adult (**I**) and fetal (**J**) heart. The size of the dots indicates the proportion of cells in the respective cell type having greater-than-zero expression of these factors, while the color indicates the average log normalized expression of these genes.

© 2021. He J et al. Published online at http://www.aginganddisease.org/EN/10.14336/AD.2021.0301

SUPPLEMENTARY DATA

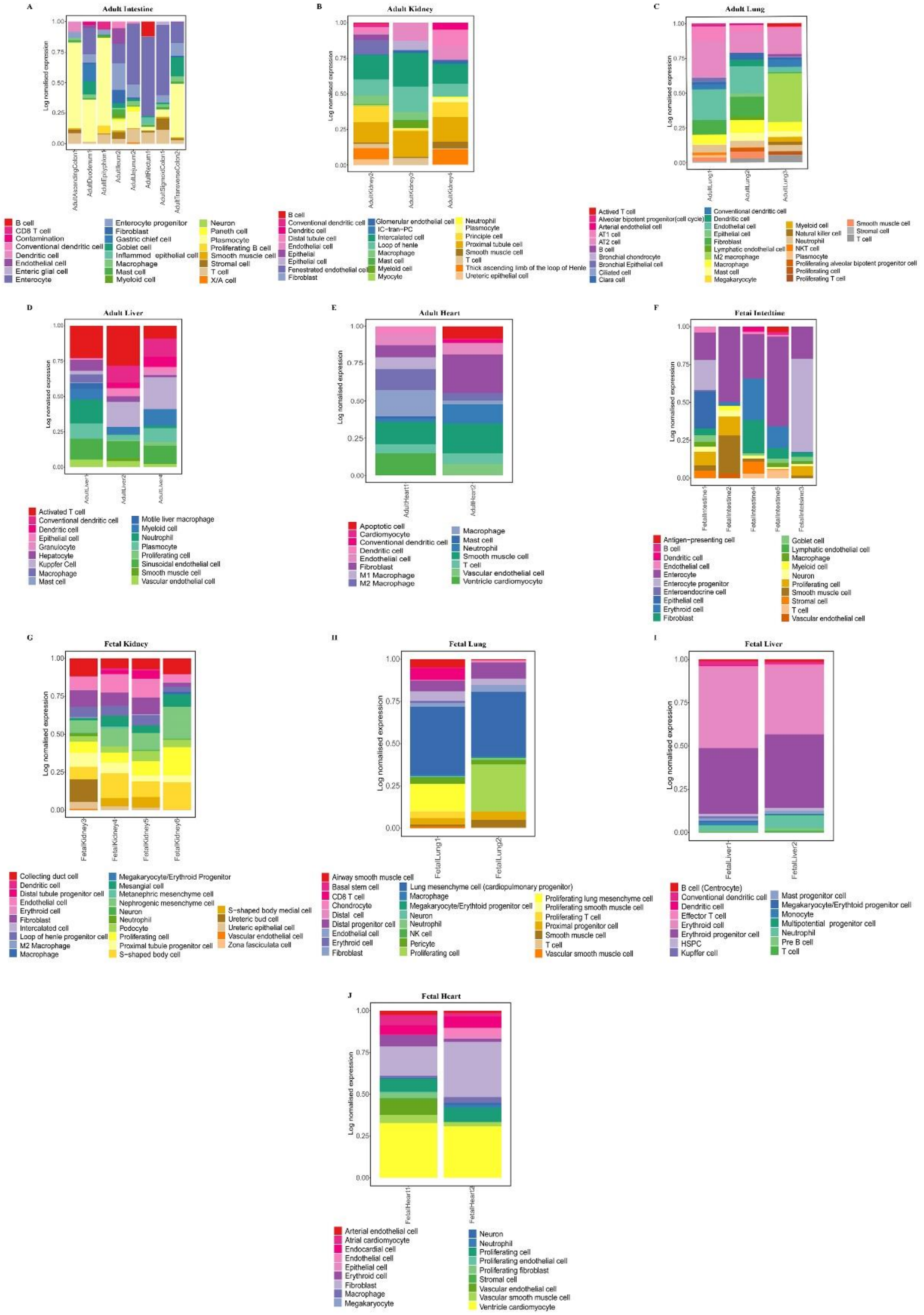

Supplementary Figure 4. Cell proportion of adult and fetal intestine (**A, F**), kidney (**B, G**), lung (**C, H**), liver (**D, I**), heart (**E, J**).

© 2021. He J et al. Published online at http://www.aginganddisease.org/EN/10.14336/AD.2021.0301

SUPPLEMENTARY DATA

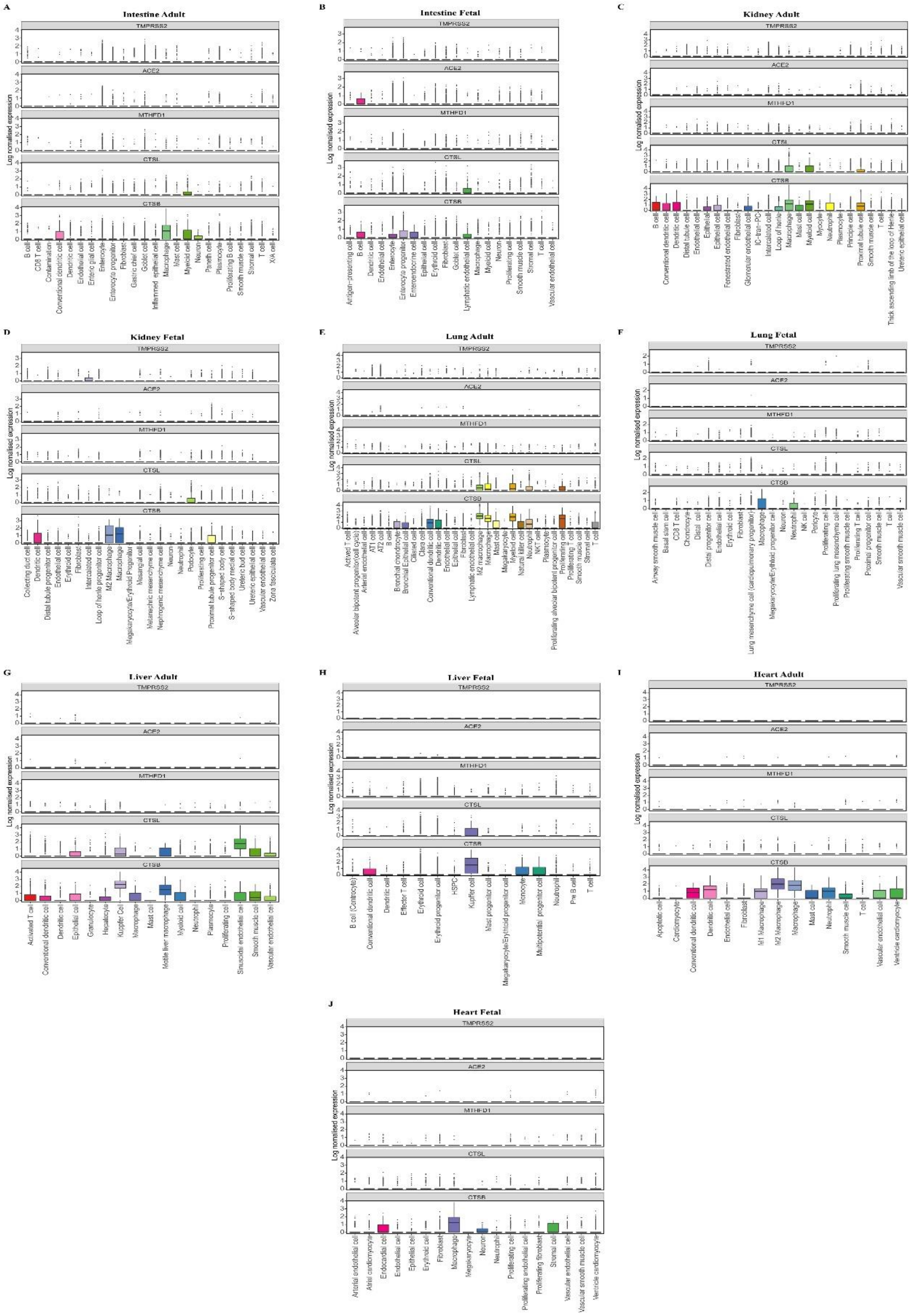

Supplementary Figure 5. Bar plot of the RNA expression level of *TMPRSS2*, *ACE2*, *MTHFD1*, *CTSL* and *CTSB* in adult (A, C, E, G, I) and fetal (B, D, F, H, J) intestine (A, B), kidney (C, D), lung (E, F), liver (G, H), heart (I, J). Raw expression values were normalized, log transformed and summarized by published cell clustering where available or reproduced clustering annotated using marker genes and cell type nomenclature from the respective studies.

© 2021. He J et al. Published online at http://www.aginganddisease.org/EN/10.14336/AD.2021.0301

SUPPLEMENTARY DATA

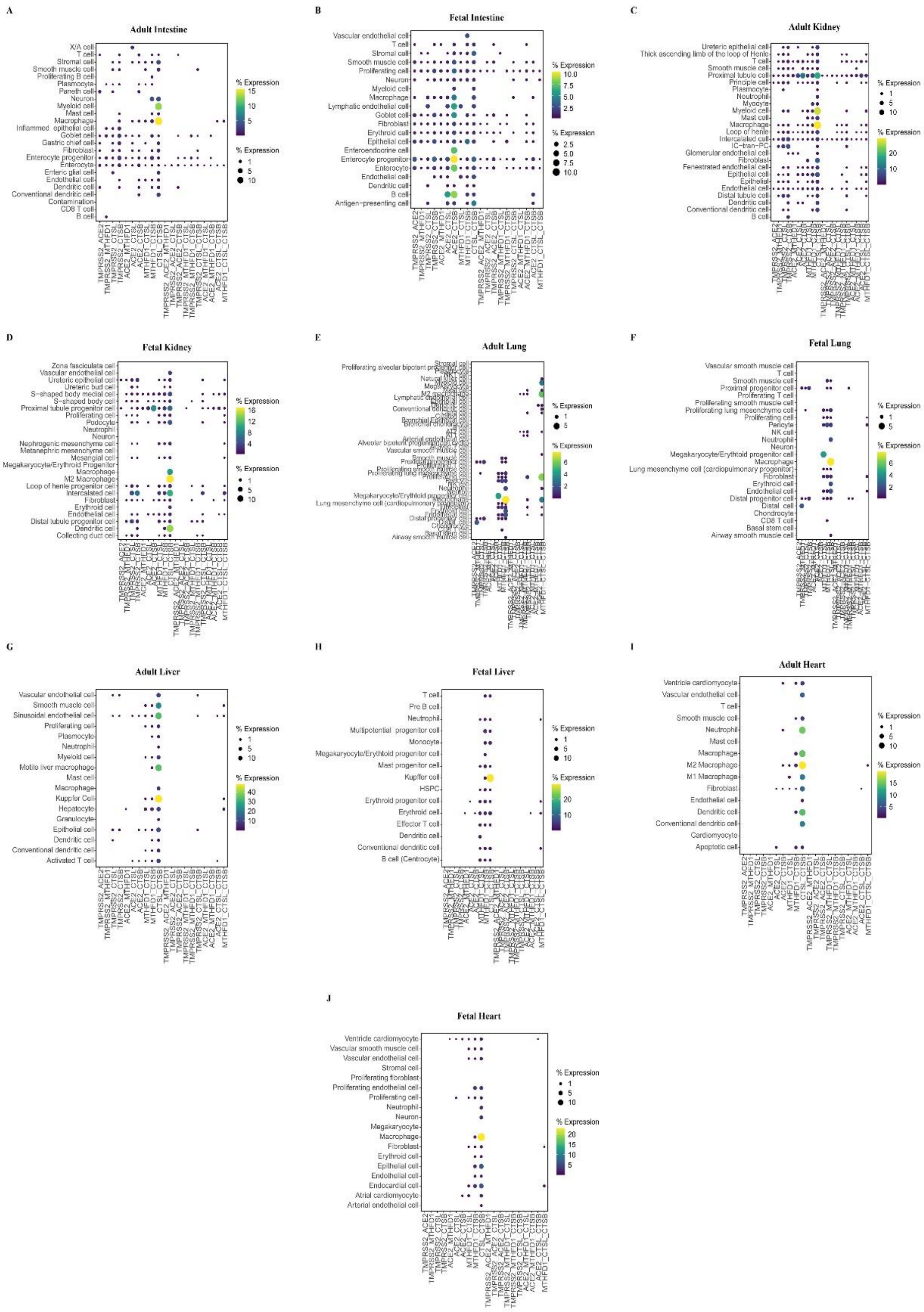

Supplementary Figure 6. The factors' co-expression from dataset of adult (C, G, K, O, S) and fetal (D, H, L, P, T) intestine (C, D), kidney (G, H), lung (K, L), liver (O, P), heart (S, T). The dot size and color represent the proportion of cells within the respective cell type co-expressing the genes.

© 2021. He J et al. Published online at http://www.aginganddisease.org/EN/10.14336/AD.2021.0301

SUPPLEMENTARY DATA

Supplementary Table 1. Basic information of samples.

	Organ	Gender	Age	Source of sample		Medical history	Sampling location
				Abortion/DCD/Operation	Cause of death		
Intestine	Adult Ascending Colon1	F	47	DCD	Cerebral hemorrhage	Hypertension	Partial tissue
	Adult Duodenum1	M	52	DCD	Traumatic brain injury		Partial tissue
	Adult Epityphlon	F	47	DCD	Cerebral hemorrhage	Hypertension	Partial tissue
	Adult Ileum2	M	41	DCD	Spontaneous brain hemorrhage		Partial tissue
	Adult Jejunum2	M	64	Operation		Cardia cancer	Proximal jejunum
	Adult Rectum1	F	47	DCD	Cerebral hemorrhage	Hypertension	Partial tissue
	Adult Sigmoid Colon1	F	47	DCD	Cerebral hemorrhage	Hypertension	Lower part of the sigmoid colon
	Adult Transverse Colon1	F	51	DCD	Respiratory failure	Severe asthma	Middle of the transverse colon
	Adult Transverse Colon2	F	83	Operation		Colon cancer	Lower colon
	Fetal Intestine1	F	12	Abortion			Intact tissue
	Fetal Intestine2	M	10	Abortion			Intact tissue
	Fetal Intestine4	F	11	Abortion			Intact tissue
	Fetal Intestine5	M	12	Abortion			Intact tissue
Kidney	Adult Kidney2	M	66	Operation		Kidney cancer	Adjacent normal tissue
	Adult Kidney3	M	41	DCD	Spontaneous brain hemorrhage		Partial tissue
	Adult Kidney4	M	57	Operation		Kidney cancer	left kidney
	Fetal Kidney3	M	13	Abortion			Intact tissue
	Fetal Kidney4	M	11	Abortion			Intact tissue
	Fetal Kidney5	M	12	Abortion			Intact tissue
	Fetal Kidney6	F	11	Abortion			Intact tissue
Lung	Adult Lung1	M	21	DCD	Traffic accident	High BMI	The lower lobe of right lung
	Adult Lung2	F	21	DCD	Traumatic brain injury		The lower lobe of right lung
	Adult Lung3	F	49	Operation		Pulmonary nodule	inferior lobe of right lung
	Fetal Lung1	F	12	Abortion			Intact tissue
	Fetal Lung2	F	11	Abortion			Intact tissue
Liver	Adult Liver1	F	21	DCD	Traumatic brain injury		Right lobe
	Adult Liver4	F	23	Operation		Hocal nodular hyperplasia	Left lateral lobe
	Fetal Liver1	F	26	Abortion			Intact tissue
Heart	Adult Heart1	M	52	DCD	Traumatic brain injury		Left ventricular apex
	Adult Heart2	F	47	DCD	Cerebral hemorrhage	Hypertension	Left ventricle
	Fetal	F	12	Abortion			Intact tissue
	Fetal	F	11	Abortion			Intact tissue
Skin	Fetal Skin2	F	7	Abortion			Limb
	Fetal Skin3	F	11	Abortion			scalp

© 2021. He J et al. Published online at http://www.aginganddisease.org/EN/10.14336/AD.2021.0301

SUPPLEMENTARY DATA

Omentum	Adult Omentum1	M	21	DCD	Brain death (traffic accident)	High BMI	Partial tissue
	Adult Omentum2	M	41	DCD	Spontaneous brain hemorrhage		Partial tissue
	Adult Omentum3	M	56	Operation		Esophageal cancer	Abdominal cavity
Placenta	Placenta1	F	10	Abortion			Partial tissue

DCD, Donation after Cardiac Death

Supplementary Table 2. Expression level of COVID-19 receptors.

**Organ**		Cluster No.	Annotation	**Organ**		Cluster No.	Annotation
**Intestine**	**Adult Ascending colon**	1	B cell (Plasmocyte)_IGLV3-1 high	**Intestine**	**Fetal Intestine 1**	1	Enterocyte_RPS2P46 high
		2	T cell			2	Enterocyte progenitor_APOA4 high
		3	Dendritic cell			3	Enterocyte_GSTA1 high
		4	**Enterocyte progenitor**			4	Proliferating cell
		5	**Fibroblast**			5	Stromal cell
		6	**Macrophage**			6	Goblet cell
		7	B cell (Plasmocyte)_IGHG3 high			7	Myofibroblast
		8	**Smooth muscle cell**			8	Endothelial cell
		9	**Mast cell**			9	Smooth muscle cell
		10	**Schwann cell**			10	Neuron
		11	**Enterocyte_CA7 high**			11	Macrophage
	**Adult_Duodenum**	1	B cell (Plasmocyte)_IGHM/HA_IGK high			12	Enteroendocrine cell
		2	Mucous neck cell		**Fetal_Intestine 2**	1	Enterocyte
		3	**Enterocyte_GSTA1 high**			2	Myofibroblast
		4	B cell (Plasmocyte)_IGH/HA_IGL high			3	Proliferating cell
		5	Goblet cell_LCN2 high			4	Neuron
		6	Enterocyte progenitor			5	Neutrophil
		7	**Enterocyte_APOA1 high**			6	Endothelial cell
		8	B cell (Plasmocyte)_IGH/HA_IGK high			7	Erythroid cell
		9	**Macrophage**		**Fetal_Intestine 4**	1	Erythroid cell_HBA2 high
		10	B cell_Contamination			2	Enterocyte _APOA4 high
		11	Endothelial cell			3	Enterocyte_AGR2 high

© 2021. He J et al. Published online at http://www.aginganddisease.org/EN/10.14336/AD.2021.0301

	12	Enterocyte_BEST4 high
	13	B cell (Plasmocyte)_IGHA_IGK high
	14	T cell
	15	Enterocyte_MT gene high
	16	**Fibroblast**
	17	**Mast cell**
	18	B cell (Plasmocyte)_IGHM/HA_IGK high
	19	**Goblet cell_CLCA1 high**
**Adult_Epityphlon**	1	B cell (Plasmocyte)_JCHAIN high
	2	Lymphocyte
	3	**T cell**
	4	**CD8 T cell**
	5	Proliferating B cell
	6	B cell (Plasmocyte)_IGHG3 high
	7	**Enterocyte**
	8	Myeloid cell
	9	B cell (Plasmocyte)_IGKV1-9 high
	10	Mast cell
	11	**Stromal cell**
	12	**Goblet cell**
**Adult_Ileum**	1	**Enterocyte**
	2	Enterocyte progenitor_REGIA high
	3	Endothelial cell
	4	Fibroblast
	5	Enterocyte progenitor
	6	Conventional dendritic cell
	7	Stromal cell_PTGDS high
	8	B cell (Plasmocyte)_IGHG/HA high
	9	T cell
	10	Mast cell
	11	Goblet cell
	12	Endothelial progenitor cell
	13	B cell (Plasmocyte)_IGHM/HA_IGL high
	14	Neutrophil
	15	Paneth cell
	4	Fibroblast_FNDC1 high
	5	Fibroblast_HMGB2 high
	6	Stromal cell
	7	Myofibroblast
	8	Dendritic cell
	9	T cell
	10	Neuron
	11	Endothelial cell
	12	Goblet
	13	Smooth muscle cell_ACTA2 high
	14	Smooth muscle cell_ACTG2 high
	15	Neutrophil
	16	T cell_CCL4 high
	17	Erythroid cell_HBA1 high
**Fetal_Intestine 5**	1	Enterocyte _APOA4 high
	2	Epithelial cell
	3	Stromal cell_CXCL 14 high
	4	Enterocyte_GSTA1 high
	5	Macrophage
	6	Proliferating cell
	7	Endothelial cell
	8	Goblet cell
	9	Stromal cell_MGP high
	10	T cell
	11	Neuron
	12	Enteroendocrine cell_CFL1 high
	13	Enteroendocrine cell_MLN high
	14	Proliferating cell
	15	Enterocyte_GUCA2A high
	16	Smooth muscle cell_MT-RNR2 high
	17	Smooth muscle_ACT2 high
	18	Paneth cell

© 2021. He J et al. Published online at http://www.aginganddisease.org/EN/10.14336/AD.2021.0301

	16	Macrophage
	17	Smooth muscle cell
	18	Neuron
**Adult_Jejunum**	1	**Enterocyte_RBP2 high**
	2	Enterocyte progenitor_OLFM4 high
	3	B cell (Plasmocyte)_IGKV4-1 high
	4	Enterocyte_APOA1 high
	5	T cell_IFNG high
	6	**B cell (Plasmocyte)_IGLL5 high**
	7	Marcophage
	8	Paneth cell_DEFA5 high
	9	T cell_GZMA high
	10	Absorptive cell
	11	Dendritic cell
	12	Goblet cell
	13	Fibroblast
	14	T cell_XCL1 high
	15	Enteroendocrine cell
	16	Mast cell
	17	Smooth muscle cell
	18	Paneth cell_REG3A high
	19	B cell (Plasmocyte)
	20	Endothelial cell
**Adult_Rectum**	1	Goblet progenitor_MT gene high
	2	B cell
	3	T cell
	4	Enterocyte_BAG3 high
	5	Enterocyte_SLC26A3 high
	6	B cell (Plasmocyte)
	7	Enterocyte_CA7 high
	8	Stromal cell
	9	Goblet cell
	10	Macrophage
	11	Schwann cell
	12	Mast cell
	13	Enterocyte_OTOP2 high
	14	Smooth muscle cell
		Enterocyte_RN7SK high
**Adult_Sigmoid Colon**	1	Goblet cell

© 2021. He J et al. Published online at http://www.aginganddisease.org/EN/10.14336/AD.2021.0301

SUPPLEMENTARY DATA

	2	T cell
	3	Stromal cell_DCN high
	4	B cell (Plasmocyte)
	5	Enterocyte progenitor
	6	Enterocyte_GUCA2A high
	7	Unknown
	8	**Schwann cell**
	9	Enterocyte_CA7 high
	10	**Macrophage**
	11	**Mast cell**
	12	**Stromal cell_CXCL14 high**
	13	Goblet cell
	14	Smooth muscle cell
**Adult_Transverse-Colon**	1	**Enterocyte progenitor**
	2	Goblet cell
	3	Enterocyte_SLC26A3 high
	4	B cell (Plasmocyte)_IGHA_IGK high
	5	B cell (Plasmocyte)_IGHA_IGL high
	6	Enterocyte_BEST4 high
	7	T cell
	8	B cell (Plasmocyte)_IGHG_IGK high
	9	**Macrophage**
	10	**Stromal cell_MGP high**
	11	**Stromal cell_MFAP4 high**
	12	**Smooth muscle cell**
	13	**Dendritic cell**
	14	**Endothelial cell**
	15	**Neutrophil**
	16	Schwann cell
**Adult_Transverse-Colon 2**	1	B cell (Plasmoyte)_IGKC high
	2	B cell (Plasmocyte)_IGLC3 high
	3	Enterocyte progenitor
	4	Goblet cell_FCGBP high
	5	Goblet cell_PIGR high
	6	Enterocyte_CA1 high
	7	Macrophage
	8	B cell (Plasmocyte)_IGHG1 high

© 2021. He J et al. Published online at http://www.aginganddisease.org/EN/10.14336/AD.2021.0301

		9	Enterocyte_SLC26A3 high				
		10	T cell				
		11	Goblet cell_ZG16 high				
		12	Enterocyte_SELENBP1 high				
		13	Enterocyte_BEST4 high				
		14	Enterocyte_PHGR1 high				
		15	Mast cell				
		16	Fibroblast				
		17	Enterocyte_CA7 high				
		18	Stromal cell				
		19	Endothelial cell				
		20	Smooth muscle cell				
**Kidney**	**Adult_Kidney2**	1	Intercalated cell _SPINK 1 high	**Kidney**	**Fetal_Kidney 3**	1	Lymphatic endothelial cell
		2	Principle cell			2	Neutrophil
		3	Proximal tubule cell_MT1G high			3	Proximal tubule progenitor cell
		4	Fenestrated endothelial cell_SELE high			4	S-shaped body cell_PSMA2 high
		5	Loop of Head (Thick ascending limb)			5	Nephrogenic mesenchyme cell_DAPL1 high
		6	Loop of Henle_SPP1 high			6	Proliferating cell
		7	Macrophage			7	Loop of Henle progenitor cell
		8	Ureteric epithelial cell			8	Interstitial progenitor cell
		9	Epithelial cell_NUPR1 high			9	Endothelial cell_PLVAP high
		10	Loop of Henle_SFN high			10	Ureteric epithelial cell
		11	Distal tubule cell			11	Interstitial cell_POSTN high
		12	Proximal tubule cell_ALDOB high			12	Podocyte
		13	T cell			13	Endothelial cell_EMCN high
		14	Conventional dendritic cell			14	Mesangial cell
		15	Fenesrated endothelial cell_EMCN high			15	Neutrophil
		16	Glomerular endothelial cell_AQPI high			16	Endothelial cell_GJA5 high
		17	Intercalated cell_SLC26A4 high			17	Neuron
		18	Myocyte			18	Erythroid cell
		19	IC-tran-PC			19	M2 Macrophage
		20	Myofibroblast			20	Endothelial cell_CCL21 high
		21	B cell		**Fetal_Kidney 4**	1	S-shaped body cell_LINC01158 high
		22	Mast cell			2	Endothelial cell_PLVAP high
	**Adult_Kidney3**	1	Proximal tubule cell_LRP2 high			3	Proliferating cell
		2	Intercalated cell_SPINK1 high			4	Proximal tubule progenitor cell
		3	Fenestrated endothelial cell_SELE high			5	Loop of Henle progenitor cell
		4	Loop of Henle_ANXA1 high			6	Proliferating cell

© 2021. He J et al. Published online at http://www.aginganddisease.org/EN/10.14336/AD.2021.0301

SUPPLEMENTARY DATA

	5	Loop of Henle_UMOD high
	6	Proximal tubule cell_MT1G high
	7	Neutrophil
	8	T cell
	9	Kidney epithelial
	10	Unknown
	11	Macrophage_APOC1 high
	12	Endothelial cell_EMCN high
	13	Loop of Henle_KNG1 high
	14	Glomerular endothelial cell_AQP1 high
	15	Macrophage_GPR183 high
	16	Intercalated cell_CALB1 high
	17	Principle cell
	18	Intercalated cell_SLC26A high
	19	Smooth muscle cell
	20	B cell (Plasmocyte)
	21	Mast cell
	22	Fibroblast
	23	Endothelial cell_COL4A1 high
	24	Neutrophil
**Adult_Kidney4**	1	Loop of Henle (Thick ascending limb)
	2	Principle cell
	3	Intercalated cell_SPINK1 high
	4	Distal tubule cell_SLC12A3 high
	5	Proximal tubule cell_ALDOB high
	6	Proximal tubule cell_MT1G high
	7	Dendritic cell
	8	Loop of Henle_SOD3 high
	9	Endothelial cell_IGFBP5 high
	10	Smooth muscle cell
	11	Proximal tubule cell_SOX4 high
	12	Endothelial cell_SLCA3 high
	13	Loop of Henle_SLP high
	14	Distal tubule cell_KLK1 high
	15	Intercalated cell_SLC26A4 high
	16	B cell
	17	Glomerular endothelial cell_AQP1 high
	7	Collecting duct cell_CALB1 high
	8	Interstitial progenitor cell
	9	S-shaped body medial cell
	10	Collecting duct cell_CRABP1 high
	11	Podocyte
	12	Nephrogenic mesenchyme cell_DAPL1 high
	13	Distal tubule progenitor cell
	14	Ureteric epithelial cell
	15	S-shaped body cell_CFAP126 high
	16	Mesangial cell
	17	Interstitial cell_PTN high
	18	Interstitial cell_POSTN high
	19	Proliferating cell
	20	Dendritic cell
	21	Endothelial cell_GJA5 high
**Fetal_Kidney 5**	1	Endothelial cell_PLVAP high
	2	Nephrogenic mesenchyme cell_DAPL1 high
	3	S-shaped body cell
	4	Proliferating cell
	5	Interstitial progenitor cell
	6	S-shaped body medial cell
	7	Collecting duct cell
	8	Loop of Henle progenitor
	9	Podocyte
	10	Distal tubule progenitor cell
	11	Collecting duct cell
	12	Proximal tubule progenitor cell
	13	Interstitial cell_POSTN high
	14	Ureteric epithelial cell
	15	Dendritic cell
	16	Neuron
	17	Endothelial cell_CCL21 high
	18	Intercalated cell
	19	Erythroid cell
**Fetal_Kidney 6**	1	Nephrogenic mesenchyme cell_DAPL1 high
	2	Proliferating cell
	3	S-shaped body cell

© 2021. He J et al. Published online at http://www.aginganddisease.org/EN/10.14336/AD.2021.0301

SUPPLEMENTARY DATA

		18	Intercalated cell_CALB1 high			4	Collecting duct cell
		19	Neutrophil			5	Interstitial progenitor cell
		20	Endothelial cell_EMCN high			6	Endothelial cell_PLVAP high
		21	Ureteric epithelial cell			7	Podocyte
		22	T cell			8	Proximal tubule progenitor cell
						9	Loop of Henle progenitor cell
						10	Interstitial cell_POSTN high
						11	Macrophage
						12	Neuron
						13	Erythroid cell
						14	Zona fasciculate cell
						15	Megakaryocyte/Erythroid Progenitor
**Lung**	**Adult_Lung1**	1	AT2 cell	**Lung**	**Fetal_Lung 1**	1	Lung mesenchyme cell
		2	Endothelial cell_TMEM100 high			2	Lung mesenchyme cell
		3	AT1 cell			3	CD8 T cell
		4	Fibroblast			4	Distal progenitor cell
		5	M2 Macrophage			5	Proliferating lung mesenchyme cell_UBE2C high
		6	Neutrophil			6	Endothelial cell_IGFBP3 high
		7	Endothelial cell_VWF high			7	Airway smooth muscle cell
		8	Conventional dendritic cell			8	Proliferating T cell
		9	Smooth muscle cell			9	Proximal progenitor cell
		10	Basal/epithelial cell			10	Pericyte l
		11	T cell			11	Fibroblast
		12	Endothelial cell_SELE high			12	Erythroid cell
		13	B cell (Plasmocyte)_IGHA/HM high			13	Myofibroblast_ELN high
		14	Alveolar bipotent/intermediate cell			14	Macrophage
		15	Fibroblast			15	Smooth muscle cell_ACTG2 high
		16	Clara cell			16	Endothelial cell_Col4A1 high
		17	Mast cell			17	NK cell
		18	Fibroblast_SFRP high			18	Chondrocyte
		19	Arterial endothelial cell			19	Neuron
		20	Clara cell_BPIFB1 high			20	Myofibroblast
		21	Chondrocyte			21	Basal/epithelial cell
		22	Ciliated cell			22	Proliferating smooth muscle cell
		23	Megakaryocyte		**Fetal_Lung 2**	1	Lung mesenchyme cell
		24	Macrophage			2	Proliferating cell_KIAA010 high
		25	B cell (Plasmocyte)_IGHG high			3	Proliferating cell_UBE2C high
	**Adult_Lung2**	1	AT2 cell			4	Distal progenitor cell

© 2021. He J et al. Published online at http://www.aginganddisease.org/EN/10.14336/AD.2021.0301

	2	Fibroblast
	3	Endothelial cell_SELE high
	4	Macrophage
	5	Endothelial cell_VWF high
	6	Mast cell
	7	AT1 cell
	8	Neutrophil
	9	Clara cell_KLK11 high
	10	Endothelial cell_TMEM100 high
	11	Smooth muscle cell_ACTA2 high
	12	Dendritic Cell
	13	T cell
	14	Lymphatic endothelial cell
	15	Proliferating alveolar bipotent progenitor cell
	16	Smooth muscle cell_COX4I2 high
	17	Monocyte
	18	Epithelial cell_PLA2G2A high
	19	Arterial endothelial cell
	20	Epithelial cell_S100A2 high
	21	Clara cell_BPIFB1 high
	22	B cell
	23	Proliferating cell
**Adult- Lung 3**	1	M2 macrophage
	2	AT2 cell
	3	Conventional dendritic cell
	4	Macrophage_CCL20 high
	5	T cell
	6	Neutrophil
	7	Mast cell
	8	Myeloid cell
	9	Actived T cell
	10	NK cell
	11	Endothelial cell_SPARCL1 high
	12	Monocyte
	13	B cell
	14	AT1 cell
	15	Proliferating cell
	16	Endothelial cell_ACKR1 high
	17	Clara cell
	18	Macrophage_VSIG4 high

5	Smooth muscle cell
6	Proximal progenitor cell_SCGB3A2 high
7	Fibroblast
8	Endothelial cell
9	Pericyte
10	Conventional dendritic cell
11	Proliferating cell
12	Proximal progenitor cell_GRP high
13	Airway smooth muscle cell
14	T cell
15	Chondrocyte
16	Megakaryocyte/Erythroid progenitor cell
17	Macrophage

© 2021. He J et al. Published online at http://www.aginganddisease.org/EN/10.14336/AD.2021.0301

SUPPLEMENTARY DATA

		19	Fibroblast				
		20	Proliferating T cell				
		21	Ciliated cell				
		22	Smooth muscle cell				
**Liver**	**Adult_Liver1**	1	Sinusoidal endothelial cell	**Liver**	**Fetal_Liver 1**	1	Erythroid cell_HBM high
		2	Activated T cell			2	Erythroid progenitor cell_REXO2 high
		3	B cell (Plasmocyte)_IGHM/HG high			3	Erythroid progenitor cell_NPM1 high
		4	Myeloid cell			4	Erythroid cell_HBB high
		5	Neutrophil_CD177 high			5	Conventional dendritic cell
		6	Macrophage			6	Neutrophil_ELANE high
		7	Hepatocyte_FGB high			7	Mast progenitor cell
		8	Vascular endothelial cell			8	HSPC
		9	Neutrophil_LCN2 high			9	Effector T cell
		10	Motile liver macrophage			10	B cell (Centrocyte)
		11	Neutropgil_CAMP high			11	Neutrophil_PRTN3 high
		12	Kupffer cell			12	Neutrophil_CAMP high
		13	Hepatocyte_GSTA1 high			13	Neutrophil_DEFA high
		14	Neutrophil_ELANE high			14	Monocyte
		15	Epithelial cell			15	Pre B cell
		16	Mast cell1			16	Unknown
	**Adult_Liver4**	1	Kupffer cell			17	T cell
		2	Conventional dendritic			18	Kupffer cell
		3	Myeloid cell			19	Multipotential progenitor cell
		4	Sinusoidal endothelial cell_FCN3 high			20	Megakaryocyte/Erythroid progenitor cell
		5	Activated T cell			21	Dendritic cell
		6	Dendritic cell				
		7	Epithelial cell				
		8	B cell (Plasmocyte)_IGHA/HM_IGK high				
		9	Proliferating cell				
		10	B cell (Plasmocyte)_IGHG_IGK high				
		11	Neutrophil				
		12	B cell (Plasmocyte)_IGHA/HM_IGL high				
		13	Vascular endothelial cell				
		14	Sinusodial endothelial cell_FCN1 high				
		15	Neutrophil				
		16	B cell (Plasmocyte)_IGHG_IGL high				

© 2021. He J et al. Published online at http://www.aginganddisease.org/EN/10.14336/AD.2021.0301

		17	Hepatocyte
**Heart**	**Adult_Heart1**	1	Smooth muscle cell
		2	Ventricle cardiomyocyte
		3	M2 Macrophage
		4	Macrophage_CCL3L3 high
		5	Fibroblast
		6	M1 Macrophage
		7	Macrophage_SPP1 high
		8	Endothelial cell
		9	T cell
		10	Dendritic cell
		11	Neutrophil
		12	Mast cell
	**Adult_Heart2**	1	Fibroblast
		2	Smooth muscle
		3	Neutrophil
		4	Smooth muscle cell_CYCS high
		5	Endothelial cell_NEAT1 high
		6	Endothelial cell_ACKR1 high
		7	T cell
		8	M2 Macrophage
		9	Neutrophil
		10	Macrophage
		11	Conventional dendritic cell
		12	Cardiomyocyte
**Heart**	**Fetal_Heart 1**	1	Ventricle cardiomyocyte_MT gene high
		2	Fibroblast_DCN high
		3	Endothelial cell_FABP4 high
		4	Ventricle Cardiomyocyte_MYL2 high
		5	Proliferating cell
		6	Erythroid cell
		7	Endocardial cell
		8	Vascular smooth muscle cell
		9	Fibroblast_PENK high
		10	Atrial cardiomyocyte_MT high
		11	Proliferating fibroblast
		12	Arterial endothelial cell
		13	Atrial cardiomyocyte_NPPA high
		14	Antigen-presenting cell
		15	Macrophage
		16	Proliferating endothelial cell
		17	Stromal cell
		18	Neuron
		19	Atrial cardiomyocyte_MYH6 high
		20	Epithelial cell
		21	Megakaryocyte
	**Fetal_Heart 2**	1	Fibroblast_DCN high
		2	Ventricle cardiomyocyte_CSRP3 high
		3	Fibroblast_PENK high
		4	Endocardial cell
		5	Ventricle cardiomyocyte_MB high
		6	Endothelial cell_FABP4 high
		7	Proliferating cell_TOP2A high
		8	Proliferating cell_PTTG12 high
		9	Macrophage
		10	Vascular smooth muscle cell
		11	Atrial cardiomycyte_NPPA high
		12	Smooth muscle cell
		13	Erythroid cell
		14	Neutrodhil
		15	Arterial endothelial cell_GJA5 high
		16	Neuron
		17	Antigen-presenting cell
		18	Epithelial cell_ITLN high

© 2021. He J et al. Published online at http://www.aginganddisease.org/EN/10.14336/AD.2021.0301
